# Gut Microbiota and Atherosclerosis—Focusing on the Plaque Stability

**DOI:** 10.3389/fcvm.2021.668532

**Published:** 2021-08-03

**Authors:** Xinyi Shen, Lihua Li, Zhen Sun, Guangyao Zang, Lili Zhang, Chen Shao, Zhongqun Wang

**Affiliations:** ^1^Department of Cardiology, Affiliated Hospital of Jiangsu University, Zhenjiang, China; ^2^Department of Pathology, Affiliated Hospital of Jiangsu University, Zhenjiang, China

**Keywords:** atherosclerosis, metabolites, therapies, plaque stability, gut microbiota

## Abstract

Cardiovascular diseases (CVDs) are major causes of mortality and morbidity in the modern society. The rupture of atherosclerotic plaque can induce thrombus formation, which is the main cause of acute cardiovascular events. Recently, many studies have demonstrated that there are some relationships between microbiota and atherosclerosis. In this review, we will focus on the effect of the microbiota and the microbe-derived metabolites, including trimethylamine-N-oxide (TMAO), short-chain fatty acids (SCFAs), and lipopolysaccharide (LPS), on the stability of atherosclerotic plaque. Finally, we will conclude with some therapies based on the microbiota and its metabolites.

## Introduction

Cardiovascular diseases (CVDs) are the most common underlying cause of death accounting for an estimated 17.8 million of 54 million total deaths (1). Atherosclerosis, determined as to be the underlying pathology of CVD, is increasing in prevalence worldwide (2). The traditional risk factors found associated with atherosclerosis include hypertension, hyperlipidemia, diabetes mellitus, obesity, and smoking (3). In recent years, the incidence of atherosclerosis has continued to increase which may indicate that these risk factors cannot fully explain the pathogenesis of atherosclerosis. There is a more urgent need to deepen our understanding of the underlying mechanisms of atherosclerosis.

With the development of technology, our knowledge of the roles of the microbiota in the host has increased drastically. With 16S RNA sequencing and high-throughput sequencing, scientists have found that in the atherosclerotic plaque, bacterial DNA exists (4). Besides, there are several studies proving that the metabolites from microbiota also can influence the development of atherosclerosis. Short-chain fatty acids have already been proven to play protective roles in atherosclerosis, stabilizing plaque (5, 6). Simultaneously, they also found that trimethylamine-N-oxide and lipopolysaccharide make the vascular endothelium disorder and plaque unstable and facilitate thrombosis, but the intrinsic mechanism is still not clear (7, 8).

In recent years, several studies have investigated the effects of the gut microbiota on atherosclerosis and proved that there are some connections between cardiovascular events and atherosclerotic plaque characteristics (9–19). However, the roles of the gut microbiota in the stability of plaque are still unclear. Therefore, in this review, we will discuss the microbiota changing in the atherosclerosis and whether there is a relationship between the bacterial flora and different conditions of atherosclerosis. Besides, we will state the metabolites from microbiota and how they can impact atherosclerosis. Moreover, we will discuss whether therapeutic strategies that target the intestinal microbiota to alleviate the development of atherosclerosis are possible.

## Microbiota

### Composition and Development of Gut Microbiota

Gut microbiota is the collection of microorganisms including viruses, archaea, fungi, and bacteria which are the most abundant components in the gut (20). The microbiota usually exists in the gastrointestinal (GI) tract, especially in the ascending colon which is mostly anaerobic and characterized by a nutrient-rich environment that is beneficial for the propagation of microorganisms (21). The infant's gut microbiota, determined by the mode of delivery, appears to be unstable and lacks diversity. The intestines of infants born through the vagina, primarily composed of *Lactobacillus* and *Prevotella*, are initially colonized by vaginal microorganisms. However, the infant's gut microbiota, born through cesarean section, is modified and dominated by *Streptococcus, Corynebacterium*, and *Propionibacterium*, similar to the skin of human (22). Thus, the infants who were delivered by cesarean section may be more susceptible to pathogen infections. Watson et al. found that 64–82% of newborns infected with methicillin-resistant *Staphylococcus aureus* were delivered by cesarean section (23). Influenced by the environment around the infant, the intestinal microbiota evolves until approximately the age of 3, becoming a diverse, complex, and stable collection, with 6070% similarity to the adult gut microbiota (24). During development, some ingredients can affect the microbiota such as diet (i.e., breast milk or formula feeding) and the use of antibiotics, which is usually reported to be related to the disruption of the infant gut microbiota. Several experiments found that the infant gut microbiota can benefit long-term health and human gut microbiota in the early stage is related to particular adult health conditions (21, 25–27).

**Figure 1:**
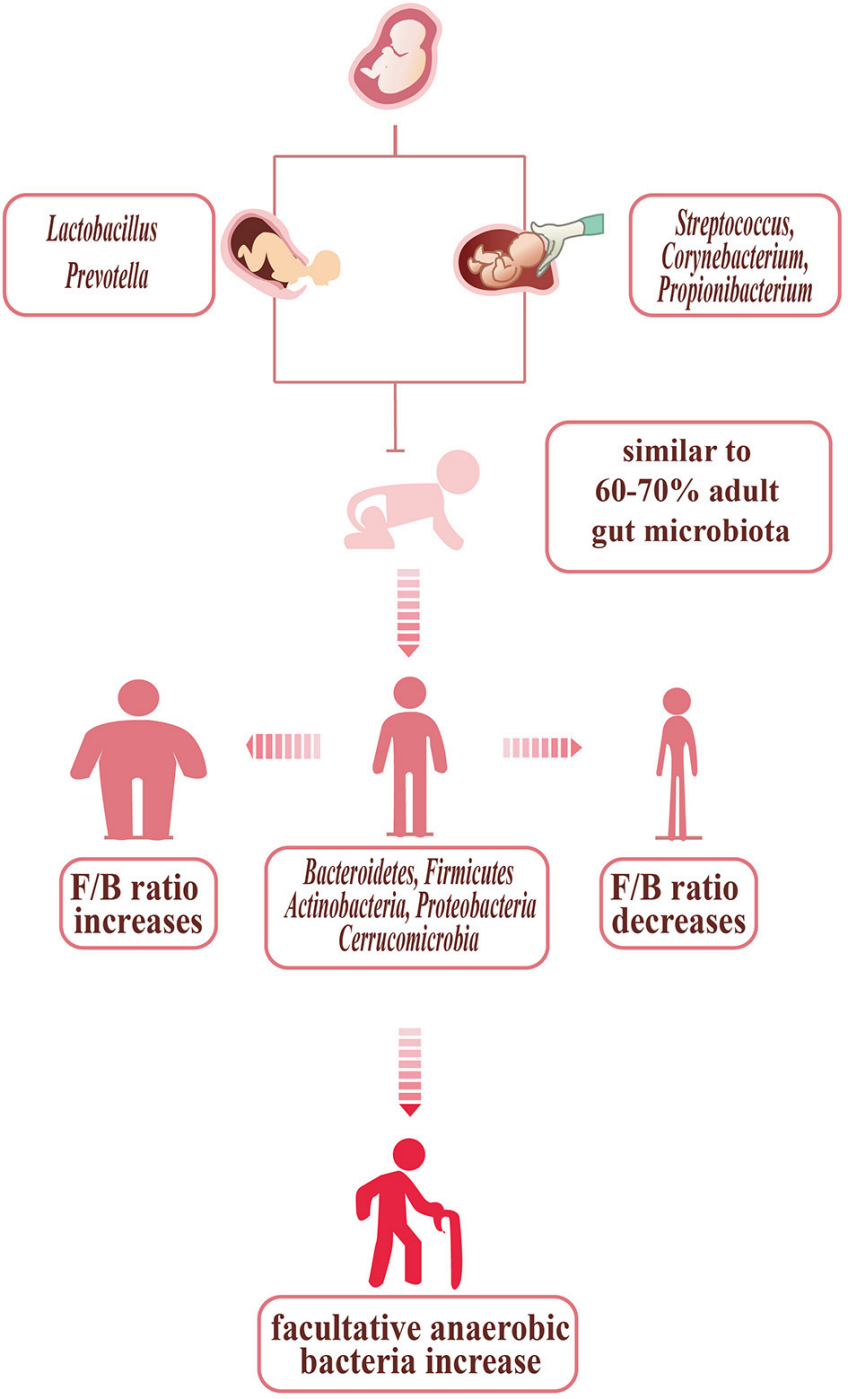
Bacterial changes with age, delivery mode, and body shape. The infants', born through vagina, are initially colonized by vaginal microorganisms—*Lactobacillus* and *Prevotella*. The infants', born through cesarean section, are dominated by *Streptococcus, Corynebacterium*, and *Propionibacterium*, similar to the skin of humans. In children up to 3 years old, the gut microbiota is stable, with 60–70% similarity to the adult gut microbiota. The gut microbiota of adult with normal shape is mainly composed of five phyla: *Bacteroidetes, Firmicutes, Actinobacteria, Proteobacteria*, and *Cerrucomicrobia*. The adults who are so slim are prone to show the F/B ratio increasing, while the F/B ratio of the obese decreasing. The gut microbiota of the elder changed: the diversity decreasing, with facultative anaerobic bacteria increasing.

The gut microbiota of adults is mainly composed of five phyla: *Bacteroidetes, Firmicutes, Actinobacteria, Proteobacteria*, and *Cerrucomicrobia*. The healthy bacterial community *Bacteroidetes* and *Firmicutes* together account for over 90% of the total bacterial species (28). Simultaneously, the *Firmicutes*/*Bacteroidetes* ratio (F/B ratio), which is viewed as a health indicator of intestinal microbiota in all individuals, in different people is not the same. The F/B ratio increases in people who are obese (the phylum *Firmicutes* increasing, with the phylum *Bacteroidetes* decreasing) and is also related to some cardiovascular diseases (29–35). Emoto et al. proved that in coronary artery disease (CAD) patients, there are some changes in microbial composition, with a significant increase in *Lactobacillales* (*Firmicutes*) abundance and a decrease in *Bacteroidetes* abundance (13). Besides, several studies in hypertensive models demonstrated that there is a higher F/B ratio (Figure 1) (31, 36, 37). The gut microbiota not only develops in the early stage of infant as mentioned above but in elderly individuals also changes within bacterial diversity and shifting of the dominant species (e.g., decreasing abundance of beneficial microorganisms and increasing abundance of facultative anaerobic bacteria). These changes may be related to the development of atherosclerosis in elderly individuals (38, 39).

### Leaky Gut and Atherosclerosis

The term “dysbiosis” usually refers to the imbalance of microbial communities, which is related to the changes in either microbial composition or mucosal barrier function disruption. Gut dysbiosis is usually caused by various factors such as high-fat diet, several diseases, and antibiotic overuse (20, 40). Gut microbiota changes can also affect gut permeability, thereby leading gut bacterial DNA translocation, influencing metabolites and endotoxins absorbed into circulation. In the healthy condition, there is a multifaceted intestinal barrier system including physical, biochemical, and immunological components. The intestinal epithelial cells (IECs), a single layer of cells, are the main part of the physical intestinal barrier, which are connected by tight junction proteins. In addition, two layers of mucus and gut commensal bacteria also serve as a physical barrier to prevent damaging luminal contents absorbed into circulation (41–43).

Hypertension and atherosclerosis have been proven above to be related to gut microbiota dysbiosis with an increase in the F/B ratio *via* productions of acetate and decreasing of butyrate. Butyrate, once proved to be the main energetic resource of IECs, is able to maintain the stability of gut barrier. High-fat intake thought as the risk factor for atherosclerosis can induce remarkable changes in gut microbiota composition (44–47). Desai et al. conducted an experiment to prove that consumption of low-fiber foods can lead to the expansion of mucus-degrading bacteria, including *Akkermansia muciniphila* and *Bacteroides caccae*, which significantly decreases the thickness of the mucus layer in mice, causing higher susceptibility to pathogens and endotoxins. Moreover, intake of a high-fat diet showed that *Lactobacillus* abundance greatly decreased and *Oscillibacter* abundance increased, which caused a significant permeability increase in the proximal colon (48). Besides, infections can play a role in regulating the mucosal barrier. *Helicobacter pylori* directly increasing epithelial permeability by redistributing the TJ protein ZO-1 may, for example, play a role in the atherosclerosis (49–51). Overall, dysbiosis of the gut microbiota induced by a number of factors can lead to leaky gut, causing the translocation of bacteria and some injurious metabolites produced by the gut microbiota and subsequently triggering a series of abnormal immune responses and the development of atherosclerosis.

### Microbiota and Atherosclerosis

In recent years, several studies have confirmed the presence of bacterial DNA in atherosclerotic plaque which may contribute to the development of cardiovascular disease (52). In addition, researchers have also found that compared with people without atherosclerosis, the patients with atherosclerosis (Table 1) showed some differences in the gut microbiota (9–19, 53). Garshick et al. donated aortas of Apoe-/- mice with atherosclerosis into normolipidemic wild-type mice, then feeding antibiotic. They found that compared with Apoe-/- mice, plaque size in Abx–WT recipient mice did not differ, but has a 32% reduction in CD68-expressing cells, suggesting that gut microbiota is a potential role of the microbiome to influence atherosclerosis inflammation (54). As shown in Table 1, two studies found that patients with coronary heart disease (CHD) or high IMT values, a marker of subclinical atherosclerosis, have greater *Firmicutes*/*Bacteroidetes* ratio, which are usually found in people who are obese and can confirm the protective effect of butyrate in the CVDs (15, 19). Simultaneously, the other two trials indicated that phyla *Escherichia* are enriched in the patients with subclinical carotid atherosclerosis (SCA) and coronary artery disease (CAD) which provide a new predictor in the development of atherosclerosis (16, 17). Ji et al. once found that *Acidaminococcus* was more abundant in the CAS patient. *Acidaminococcus* was once often enriched in patients with several inflammatory diseases and positively correlated with a proinflammatory diet, which may indicate that *Acidaminococcus* was a proinflammatory microbiota and represent inflammatory status in the development of AS (18). In atherosclerotic plaques, phylum *Proteobacteria* dominated and the phylum *Firmicutes*, predominantly found in the gut, is also present in atherosclerotic plaques. However, according to previous studies, there is still no conclusion on whether the microbiota is important in the development of AS or not. Mitra et al. proved that the gut microbiota in patients with stable plaques shows a significant difference from that in patients with unstable plaque. However, Hållenius et al. found that there were no major differences in bacterial DNA content or microbial composition between stable and unstable plaques (9, 11). Some researchers have observed that bacterial DNA may trigger macrophages thereby activating the innate immune system *via* Toll-like receptor 2 (TLR2) and TLR4 which are closely related to the stability of plaques (55–57). The research conducted by Chen et al. proved that the feasibility of remodeling of the gut microbiome to prevent the onset and progression of atherosclerosis in LDLr-/- mice which indicates that the gut microbiota plays some roles in atherosclerosis and may also provide a new therapy (58). One study (6) also found that introducing the proinflammatory Casp1-/- microbiota into Ldlr-/- mice promotes plaque growth with neutrophil accumulation in plaques and a significant reduction in the short-chain fatty acids producing taxonomies (*Akkermansia, Christensenellaceae, Clostridium*, and *Odoribacter*). In summary, there is no clear conclusion on whether there exists a crucial gut microbiota for the development of atherosclerosis. Studies should still focus on the items about the relationships of the gut microbiota, bacteria in plaques, and atherosclerosis.

**Table 1:** Different kinds of bacteria present in the plaque, and the gut bacterial changes between the controls and patients.

	**References**	**Year (species)**	**Method**	**Disease**	**Bacteria**
Plaque	Koren et al., (53)	2010 (H)	16SrRNA	AS	*Proteobacteria* and *Firmicutes*
					Three OTUs -*Propionibacterineae*, one OTU -*Burkholderia* were specific for atherosclerotic plaques
	Mitra et al., (9)	2015 (H)	16S rRNA	Symptomatic AS	*Helicobacteraceae, Neisseriaceae*, sulfur-consuming families and *Thiotrichaceae*
				Asymptomatic AS	*Porphyromonadaceae, Bacteroidaceae, Micrococcaceae*, and *Streptococcaceae*
	Ziganshina et al., (10)	2016 (H)	16S rRNA	AS	*Proteobacteria* (71.4–97.3%) [*Burkholderiales* (67.0–94.1%); *Actinobacteria* (0.2–24.1%) ]
					*Bacteroidetes* (0.3–2.1%) [*Saprospirales* (0.3–2.1%) and *Firmicutes* (0.1–5.2%)]
	Lindskog Jonsson et al., (11)	2017 (H)	16S rRNA	AS	*Proteobacteria* (48.3%), *Actinobacteria* (40.2%), *Firmicutes* (4.0%), *Cyanobacteria* (3.9%), *Bacteroidete*
					No differences in the microbial composition between symptomatic AS and asymptomatic AS
Gut	Koren et al., (53)	2010 (H)	16S rRNA	AS	No significant difference between the AS and controls
	Karlsson et al., (12)	2012 (H)	Metagenomics	Symptomatic AS	*Collinsella* dominating,the *Ruminococcus* enterotype genes in the peptidoglycan pathway were enriched
				control	*Eubacterium* and *Roseburia* and three species of *Bacteroides* dominating
	Emoto et al., (13)	2016 (H)	16S rRNA	CAD	The order *Lactobacillales* increasing
					Phylum *Bacteroidetes* (*Bacteroides* + *Prevotella*) decreasing
	Jie et al., (14)	2017 (H)	Metagenomics	ACVD	*Escherichia coli, Klebsiella spp., Enterobacter aerogenes, Ruminococcus gnavus, Eggerthella* lent increasing
					*Roseburia* intestinalis and *Faecalibacterium* cf. prausnitzii (butyrate-producing bacteria) reducing
	Cui et al., (15)	2017 (H)	Metagenomics	CHD	*Phyla Bacteroidetes* and *Proteobacteria* decreasing
					*Phyla Firmicutes* and *Fusobacteria* increasing
	Zhu et al., (16)	2018 (H)	16S rRNA	CAD	*Escherichia-Shigella*; *Enterococcus* enriching
					*Faecalibacterium*; *Subdoligranulum*; *Roseburia*; *Eubacterium rectale* depleting

*In plaque associated with atherosclerosis, the phylum Proteobacteria dominated and the phylum Firmicutes was also present. In the gut, we may find greater Firmicutes/Bacteroidetes ratio and phyla Escherichia was enriched in patients with atherosclerosis*.

## Metabolite

### TMAO

#### TMAO Metabolism

Trimethylamine N-oxide (TMAO), a proatherogenic metabolite, is generated from phosphatidylcholine, carnitine, γ-butyrobetaine, betaine, and crotonobetaine, which are mainly from animal-derived foods, such as red meat, eggs, dairy products, and salt-water fish (59, 60).

Trimethylamine (TMA) is generated by the enzymes produced by the gut microbiota, which includes three types. The first one is cutC/D, which is abundant in the *Desulforibrio, Desulfuricans, Protebacteria* (*Grammaprotebacteria, Deltaprotebacteria*), and *Proteus mirabilis Firmicattes* (*Clostridia, Bacillus*) (61, 62). Wang et al. have proved that 3,3-dimethyl-1-butanol (DMB), a structural analog of choline, can inhibit cutC/D choline TMA lyase activities to decrease the serum TMAO level, which may serve as a potential therapeutic approach for the treatment of cardiometabolic diseases (63). However, Orman et al. have argued that DMB has no use in inhibiting choline-metabolizing enzyme cutC. In addition, a small molecule, betain aldehyde, can effectively inhibit this kind of enzyme. The others are CtnA/B and Yea X/Y, which are proved to be highly homologous (64, 65).

TMA formed in the gut is absorbed into the portal circulation and then oxidized to TMAO by the action of hepatic flavin containing monooxygenases FMO3 and FMO1 (66). Some studies have demonstrated that knockdown of the *FMO3* gene can significantly reduce the production of TMAO (67, 68). In recent years, several new approaches have been found to inhibit the TMAO formation to alleviate the development of atherosclerosis (69, 70). However, most researches conducted *in vivo* studies are in murine models, with few in humans. In that circumstance, reducing TMAO to reduce the incidence of atherosclerosis with some effective, safe strategies may significantly benefit public health.

#### TMAO and Plaque Stability

**Figure 2:**
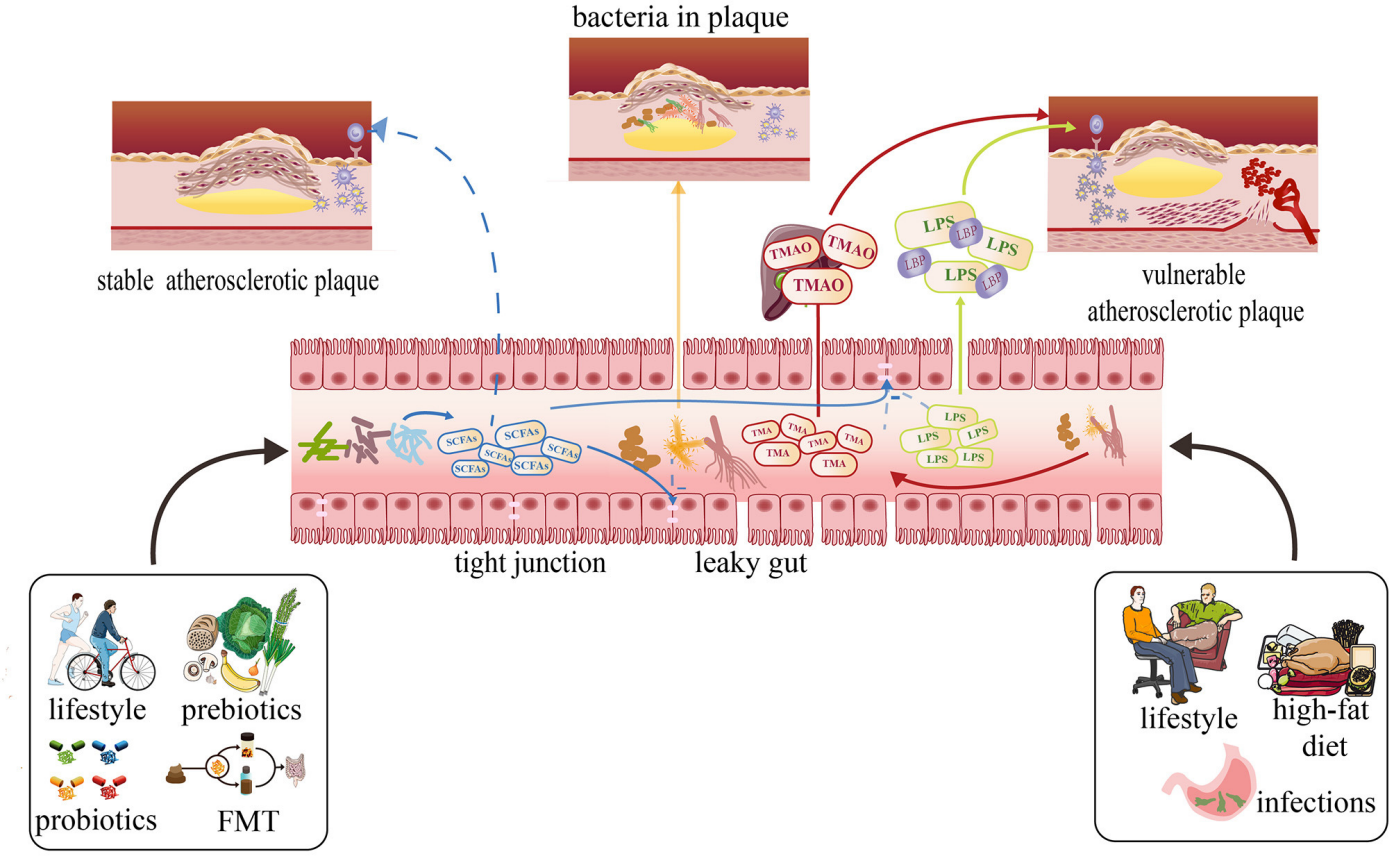
Effects of metabolites produced by the gut microbiota on atherosclerosis and possible therapies focusing on the gut microbiota to prevent atherosclerosis development and stabilize plaque. A healthy lifestyle usually can cause SCFAs to increase with related bacteria increasing. The SCFAs can protect gut barrier by supporting tight junctions to inhibit translocation of LPS, TMAO, and other damaging metabolites into the circulation. They also can prevent the development of atherosclerosis and stabilize the plaque. The high-fat diet, sedentary lifestyle may cause the plasma concentrations of TMAO and LPS to increase by increasing some kinds of bacterium and intestinal permeability, which can make the plaque more vulnerable.

Some studies have indicated that TMAO is an independent predictor of the cardiovascular diseases. In this review, we will discuss the effect of TMAO on the atherosclerotic plaque stability (Figure 2). Senthong et al. and Sheng et al. have discovered that plasma TMAO levels are closely related to the coronary atherosclerotic burden in patients with ST-segment elevation myocardial infarction (STEMI) and stable coronary artery disease (CAD), as quantified by the Synergy Between PCI with Taxus and Cardiac Surgery (SYNTAX) score (71, 72). Liu et al. once proved that the non-culprit plaques in CAD patients with higher TMAO levels have exhibited more vulnerable characteristics: thinner fibrous cap thinner (FCT), higher presence of a thin-cap fibroatheroma (TCFA), and microvessels (73). Simultaneously, in one experiment, researchers have discovered that high-choline diet fed on mice intends to increase intraplaque hemorrhage instead of altering atherosclerotic burden or plaque composition, which is a relatively novel finding (74). In recent years, more trials found that TMAO may become a totally new marker to predict future risk of CVDs, which focused on the appropriate serum levels to diagnose CVDs (75, 76). It is important to differentiate between plaque rupture and plaque erosion to determine a personalized treatment strategy. In patients with STEMI, the treatment used for plaque rupture and plaque erosion is completely different. Tan et al. have concluded that the cutoff threshold of TMAO was 1.95 μM for discriminating plaque rupture from plaque erosion with maximum sensitivity and specificity (72). In addition, TML (a precursor of TMAO) and GBB (an intermediate product of l-carnitine) were shown to predict the cardiovascular diseases independently in recent studies (77, 78).

#### Mechanisms

Mechanisms on how TMAO is able to promote atherosclerosis have already been studied at the molecular level. In the context of immunity, TMAO could activate heat shock protein 60 (HSP60), which has been shown to be the initiating event in the atherosclerosis and to take part in foam cell formation *via* Toll-like receptors, which can also be activated by SR-A1 and CD36 in macrophages after TMAO stimulation (79–82). Besides, TMAO could induce NLRP3 activation, which promotes IL-18 and IL-1β expression to trigger inflammation, which contributed to the endothelial injury that initiates atherosclerosis (83, 84). In the inflammation part, TMAO could induce the expression of IL-1, TNF-α, C-reactive protein (CRP) *via* mitogen-activated protein kinase (MAPK), and NF-kappa B (NF-κB) signaling to promote the formation of atherosclerosis (85–88). TMAO also can reduce the reverse cholesterol transport through activating the nuclear receptor Farnesoid X receptor (FXR) and small heterodimer partner (SHP) to inhibit the expression of Cyp7a1, which could reduce the synthesis of bile acids in the liver with the result of accelerating atherosclerosis development (89, 90).

### Lipopolysaccharide

Lipopolysaccharide (LPS) which is a central component of the outer membrane in Gram-negative bacteria usually exists in the gut and oral cavity has a great impact on the atherosclerosis. LPS existing in the outer membrane of Gram-negative bacteria contains three parts: lipid A, a core oligosaccharide, and the O-antigenic polysaccharide (91–94). LPS can be detected in healthy human's plasma at low concentrations (between 1 and 200 pg/ml), which proves that small amounts of LPS are able to cross the intestinal barrier. Although the function of low circulatory levels of LPS is still unclear, some researches have recently shown that it may be related to the immune modulation with some benefits on resisting infection or some damages to increase inflammation (95–97).

However, in some conditions, intestinal permeability can be increased leading the circulatory levels of LPS to rise. In atherosclerosis, the number of butyrate-producing bacteria decreased, causing a reduction of butyrate levels to result in the dysfunctional gut mucosal barrier which may cause more LPS entering into circulating blood. Besides, the high-fat diet can also lead concentrations of LPS to increase through different ways, especially through the increasing gut permeability (98, 99). Firstly, it can cause the excessive chylomicron to form, which may increase the local pressure and cause the loosening of junctional complexes between the enterocytes (100, 101). The composition of gut microbiota also can be altered due to the high-fat diet. The abundance of Gram-negative bacteria (*Bacteroides*) increases degrading mucus glycoprotein and leading to increased circulatory concentrations of toxins. Meanwhile, the abundance of Gram-positive bacteria usually promotes a stable environment and inhibits the translocation of bacteria and toxins to decrease (102, 103). Besides, the high-fat diet could decrease the activity of intestinal alkaline phosphatase, which may decrease LPS degradation to increase circulating LPS levels (104, 105). Except for the atherosclerosis and high-fat diet, the other risk factors, such as insulin resistance, hypertension, liver diseases, and other neurological diseases, may also cause the intestinal permeability to increase—leading the circulatory levels of LPS to rise (41).

#### LPS and Plaque Stability

LPS has already been proven to be involved in infectious diseases such as septic shock for several years. However, in recent years, some researches have demonstrated that there may be some relationships between LPS and atherosclerosis (106–108). Carnevale et al. have found that circulating levels of LPS and zonulin which is a protein that reflects the condition of intestinal permeability are much higher in patients with critical stenosis of carotid artery (>70%) than controls without the plaque, revealing that circulatory levels of LPS may be positively correlated with the atherosclerosis. Besides, the authors discovered that in the area of carotid plaque sections positive for LPS and TLR4, macrophages, which are closely related to the formation of atherosclerosis and the stability of the atherosclerotic plaque, were much bigger than macrophages in the area with less reactivity to LPS and TLR4. In that research, they also found that LPS can activate monocyte *via* TLR4 activation with an intracellular signaling mechanism involving expressions of Nox2 to increase, which is among the most important cellular producer of reactive oxygen species. As a result, this downstream effect can lead to the formation of oxidized LDL, proving that LPS is a molecule that promotes oxidative stress at the site of atherosclerotic plaque (Figure 2), which may lead to the rupture of the plaque (93). Yoshida et al. have detected species with differential abundance between CAD and controls with 16S ribosomal RNA gene sequencing, revealing a significantly lower abundance of *Bacteroides vulgatus, Bacteroides dorei*, and higher LPS concentration in patients with CAD. Atherosclerosis-prone mice fed with *B. vulgatus* and *B. dorei* then showed attenuation of atherosclerotic lesion formation and decrease in gut microbial lipopolysaccharide production. In that circumstance, they discussed that pathogenesis of atherosclerosis alleviation may be due to dampening of systemic innate immune cell activation and Th1-driven inflammation caused by the live Bacteroides treatment-induced reduction in plasma LPS concentration (8). Loffredo et al. have demonstrated that patients with peripheral arterial disease (PAD) have increased systemic LPS concentrations that inversely correlate with the ankle brachial index (ABI) which indicated that LPS can be the promoter of atherosclerotic burden (109). In the animal experiments, Jaw et al. found that the plaques of mice exposed in LPS presented features of vulnerability including hemorrhage and thrombus formation, which can easily induce acute plaque rupture (110).

#### LPS-Binding Protein

When LPS enters into the circulatory system, it can initiate various signaling pathways to recruit inflammatory cells into atherosclerotic lesions, which involves a large number of proteins, such as LPS-binding protein (LBP), CD14, Toll-like receptor-4 (TLR4), and MD-2. LBP, a 50-kDa polypeptide mainly synthesized in the liver and released into the bloodstream after glycosylation, is the first protein to bind with LPS, which indicates that it might be a reliable biomarker that predicts the activation of innate immune responses (111–114). Lepper et al. once found that serum LBP levels significantly increased in patients with CAD according to CAD severity compared with those without CAD, which may indicate that LBP might be able to be used as a biomarker for coronary artery disease. They also pointed that LBP deposited in regions of atherosclerosis, indicating it may be involved in the development and progression of atherosclerotic lesions (115, 116). Serrano et al. discovered that circulating LBP was positively associated with atherosclerotic risk factors, such as obesity, insulin resistance parameters, and so on. Besides, they also found that circulating LBP may independently contribute to the presence of carotid plaque and the carotid intima-media thickness, which may indicate that LBP is associated with the development of atherosclerotic lesions (117).

### Short-Chain Fatty Acids

Depending on the length of the carbon chain, the fatty acids has been classified into three types, which contains long-chain fatty acids (LCFAs, the number of carbon chain more than 12), mid-chain fatty acids (MCFAs, the number of carbon chain between 6 and 12), and short-chain fatty acids (SCFAs, the number of carbon chain <6) (118). The short-chain fatty acids are mainly produced from bacterial fermentation of food fiber and the non-digestible carbohydrates containing resistant starch (RS), non-starch polysaccharides (NSP), oligosaccharides (prebiotics), and so on. The SCFAs were mainly fermented in the large intestine due to its environment (warm, moist, anaerobic, and full of feed residues) which caters for the conditions to make prolific bacterial growth (119, 120). SCFAs which are thought as the energy resources are found to play key roles in meditating gut epithelial and immune regulation. As described above, the structure of gut epithelial covered and protected by a mucus layer is essential to prevent the bacteria from spreading into the circular blood, leading susceptibility to enhance infections and the development of chronic inflammatory diseases. In addition, the mucus layer covering and protecting the gut epithelium is maintained by the gut microbiota and diet that is mainly due to SCFAs. The main components of SCFAs were acetate, propionate, and butyrate, which account for almost 95% SCFAs. These SCFAs were mostly produced by some families of bacteria, including anaerobic *Bacteroides, Bifidobacterium, Eubacterium, Streptococcus*, and *Lactobacillus* (Table 2). The SCFAs were released at high concentrations in the ascending colon, with a decline in the transverse colon and the descending colon (121, 122).

**Table 2:** Pathways of main SCFA production and their related microorganisms.

**Type**	**Structures**	**Pathway**	**Microorganisms**	**References**
Acetate	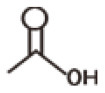	From pyruvate in acetyl-CoA pathway	*Akkermansia muciniphila, Bacteroides spp., Bifidobacterium spp.* *Prevotella spp., Ruminococcus spp*.	Koh et al., (120)
		Wood-Ljungdahl pathway	*Blautia hydrogenotrophica, Clostridium spp., Streptococcus spp*.	
Propionate	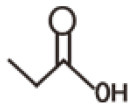	Succinate pathway	*Bacteroides uniformis.;Bacteroides vulgatus.;Prevotella copri.;* *Alistipes putredinis.;Dialister invisus;Phascolarctobacteriumsuccinatutens;* *Akkermansia muciniphila*	Louis et al., (121)
		Acrylate pathway	*Coprococcus catus*	
		Propanediol pathway	*Roseburia inulinivorans;Eubacterium hallii;Blautia obeum;*	
Butyrate	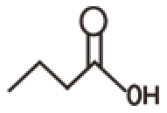	Butyryl-CoA transferase: acetate Co-A pathway	*Eubacterium rectale;Roseburia inulinivorans;Roseburia intestinalis;* *Eubacterium hallii; Anaerostipes hadrus;Coprococcus catus;* *Faecalibacterium prausnitzii;Eubacterium biformec*	Louis et al., (121)
		Butyrate kinase pathway	*Coprococcus eutactus;Subdoligranulum variabile*	

#### SCFAs and Atherosclerosis

Ghosh et al. have proven that attributing to butyrate, whole-milk consumption was inversely associated with CAC progression, which is the independent risk factor for atherosclerosis (123). One experiment once suggested that after ApoE-/- mice consumed a diet containing 1% butyrate for 10 weeks, atherosclerosis in the aorta was reduced by 50%, with lower macrophage infiltration and increased collagen deposition, suggesting a more stable fibrous cap. These phenomena are mostly associated with CD36 decreasing in macrophages and endothelial cells, reduction of proinflammatory cytokines, and lower NFkB activation. They also found that the decrease in macrophage may be due to the reduction of oxidative stress related to NADPH oxidase and iNOS expression levels (124). Vascular smooth muscle cell (VSMC) proliferation is considered to play an important role in the pathogenesis of atherosclerosis (Figure 2). There are several studies proving that butyrate can prevent the development of atherosclerosis by proliferation of VSMCs (125–127). Kasahara et al. put forward a new axis Roseburia-fiber-butyrate with atherosclerotic germ-free ApoE-/- mice colonized with a defined community of eight bacteria with or without *Roseburia intestinalis*. With that axis, the atherosclerotic plaque sizes are reduced by 30% without affecting the levels of cholesterol and TMAO perhaps relating to the ability of SCFAs to maintain the integrity of the gut (128, 129). Besides, Bartolomaeus et al. have found that propionate application to ApoE-/- mice reduced vascular inflammation and atherosclerotic lesion burden and alleviated the level of blood pressure, which is considered traditional risk factors of plaque rupture (130). Overall, the above studies may lead to the development of a new approach—supplementing SCFAs to prevent the development of atherosclerosis and stabilize the plaque in cases of the actual cardiovascular accidents. However, few studies have been conducted in humans, so more explorations of the function of SCFAs are still needed.

### Other Metabolites

There still exist other metabolites produced by gut microbiota affecting the stability of atherosclerosis. Uremic toxins, usually increasing in the chronic kidney disease (CKD), are metabolites of amino acids produced by gut microbiota (131, 132). Several studies have proved that the protein-bound uremic toxins can significantly induce VSMC proliferation and VSMC calcification, which are closely related to atherosclerosis (133–135). Besides, phytoestrogens, anthocyanins, and bile acids are also associated with atherosclerosis developing (136, 137). Recently, Nemet et al. have found a new metabolite, PAGln, generated by gut microbiota which is connected to cardiovascular disease risk because it increases the possibility of thrombus formation and thrombosis potential (138). Thus, it still has a great potential to explore whether there exist known metabolites or new metabolites connected to the gut bacteria and there being some connections between them and atherosclerosis.

## Therapy

### Daily Lifestyle, Gut Microbiota, and Atherosclerosis

#### Diet

Diet (139, 140) has shown to be closely associated with the composition and diversity of gut microbiota (Figure 2). De Filippo et al. once conducted a study that aimed to find whether there are some differences between the gut microbiota of children (BF) fed with traditional rural African diet, characterized with low in fat and animal protein and rich in starch, fiber, and plant polysaccharide and children (EU) eating a typical western diet high in animal protein, sugar, starch, and fat and low in fiber. AS a result, the *Bacteroidetes* dominated the gut microbiota of BF children, while the gut microbiota of the EU children was dominated by *Firmicutes* (141). The F/B ratio above proved to be related to the metabolic situation that is usually found increasing in people who are obese.

WHO has declared that a healthy diet comprises low saturated fats, low salts, and high fruits and vegetables (142). In recent years, scientists have found that adhering to the Mediterranean diet (MeDiet) greatly has been strongly linked to a significant reduction in overall mortality and morbidity, providing a new management approach to prevent cardiometabolic diseases. This diet is featured by high consumption of fruits, vegetables, legumes, unrefined cereals, and nuts; moderate consumption of fish, poultry, and dairy products (principally cheese and yogurt); low consumption of red meat products; use of olive oil as the main edible-fat source; and regular but moderate wine consumption (143–147). Baragetti et al. once found that people without subclinical carotid atherosclerosis consume (SCA) higher amounts of cereals, starchy vegetables, milky products, yogurts, and bakery products compared with those with SCA consuming more mechanically separated meats. The diet whereby people without SCA consume may closely in accord with MeDiet and cause changes in specific bacterial species (demonstrated above) (17). Several studies have noticed that the level of fecal SCFAs proved to play protective roles in the development of AS increased in the patients with MeDiet (148–150). Nagpal et al. have detected that MeDiet could make some influence on gut microbiota. Compared with those having the Western diet, macaques fed with the MeDiet presented higher microbial diversity and lower *Firmicutes* and *Verrucomicrobia* (lower *Firmicutes* to *Bacteroidetes* ratio) (151). One study also found that high adherence to Mediterranean diet was associated with increases in *Prevotella* and *Firmicutes*, besides low adherence was correlated with higher urinary TMAO levels (152).

Several researchers have proven that insisting on MeDiet can prevent atherosclerosis development. Some studies focused on the endothelial dysfunction which was the crucial step in AS development (153–155). Besides, adherence to Mediterranean diet also can modulate intima-media thickness and atherosclerotic plaques (156–159). Some scientists have found that MeDiet acted on the atherosclerotic inflammatory process by downregulating proinflammatory biomarkers and upregulating biomarkers associated with the stability of plaque. On the vessel imaging, people intervening with a MedDiet showed a reduction in the height of plaque, suggesting that plaques have been stabilized. In addition, a report indicated that greater adherence to MeDiet may be associated with a decreased burden of carotid atherosclerotic plaque with a reduction in plaque thickness and area. However, the mechanisms that underlie the MeDiet acting on atherosclerosis is still unknown. As we demonstrated before, MeDiet can alter the gut microbiota. Therefore, we hypothesize that the role of MeDiet in AS may have some relationships with gut microbiota.

#### Prebiotics

In the past few years, a number of scientists have discovered that intake of appropriate prebiotics may improve health and protect the cardiovascular system (160). The latest concept of prebiotics is “selectively fermented ingredients that allow specific changes, both in the composition and/or activity of the gastrointestinal microbiota that confer benefits upon host well-being and health” (161, 162). Prebiotics, including the fructans, inulin and oligofructose, resistant starches, and so on, have been found to increase the relative abundance of *Bifidobacteria* which reflects the situation of gut health to some degree (163–165). Besides, prebiotics were also able to modify blood lipid profiles mainly because of their ability to bind cholesterol or BA in the upper gut and increase sterol excretion, or for some, through their gel-forming nature, which causes a bulking effect in the intestine and triggering satiety (166–168).

#### Exercise

It has already been proven by several studies that exercise can alleviate the development of atherosclerosis (169–173). However, whether there are some relationships between the change of gut microbiota induced by sports and atherosclerosis is still not clear (Figure 2). Some investigations have found that changes in microbial diversity caused by exercise are able to improve tissue metabolism, cardiorespiratory fitness, and insulin resistance that can effectively prevent the cardiovascular incidents (174–177). Scientists discovered that compared with sedentary controls, women completing over 3 h of exercise per week had increased levels of butyrate-producing microbiota, *A. muciniphila* especially associated with a lean body mass index (BMI), and improved metabolism (178).

The modifications of gut microbiota induced by physical exercise are due to the gut transit time, the modification of the bile acids profile, the production of SCFAs *via* AMPK activation, the modulation of the Toll-like receptor (TLR) signaling pathway, immunoglobulin A, the number of B, and CD4+ T cell (179–182). These factors listed above have already been discovered to be related to the progress of atherosclerosis, providing a new approach to management. Although increasing studies have pointed that physical exercise can make some benefits on modulation of gut microbiota, the intrinsic mechanism should still be explored.

#### Others

Except for the lifestyles demonstrated before, there are several new findings about daily intervening factors of gut microbiota in enhancing the stability of plaques. As we all know, Pu-erh tea displays cholesterol-lowering properties, recent years scientists have some new discoveries on it. Xiao et al. have found that ApoE-/- mice consuming Pu-erh tea can reduce early fatty streak formation and the advanced fibrofatty plaque sizes through alleviating the chronic inflammatory state by reducing NF-κB activation and promoting macrophage apoptosis (183). In addition, Huang et al. have indicated that Pu-erh tea alters the gut microbiota in mice and humans, predominantly suppressing microbes associated with bile-salt hydrolase (BSH) activity. This finding may present a new kind of therapy on antihypercholesterolemia and antihyperlipidemia with decreasing intestinal BSH microbes or FXR-FGF15 signaling (54). Huang et al. have demonstrated that glycoursodeoxycholic acid (GUDCA) once proven to be able to improve metabolic disorders may protect against atherosclerosis progression by reducing the plaque area and elevating plaque stability. Besides, they also found that the mice gut microbiota dysbiosis fed with GUDCA administration could be partially normalized, suggesting that GUDCA is a potential approach to prevent atherosclerotic cardiovascular diseases (184). Besides, in recent years, people facing more stress and lack of sleep may not only increase inflammation-associated microbial members but also lose species that secrete anti-inflammatory metabolic products. As we described above, such conditions can accelerate the development of atherosclerosis and make plaques more prone to rupture.

### Probiotics and Atherosclerosis

The definition of probiotics is “live strains of strictly selected microorganisms which, when administered in adequate amounts, confer a health benefit on the host (185).” Probiotics can interact with the existing microbial community dynamic through competition with pathogens (Figure 2). Probiotics must satisfy the following criteria: (1) be live microorganisms; (2) can keep alive and stable before use; (3) resistant to the digestion process; (4) be scientifically proven to be beneficial to the host; and (5) be proven to be safe and reliable or a member of the original intestinal microflora. In fact, many products (e.g., yogurt) sold on the market do not cater these basic standards. Up to now, probiotics includes the related bacteria of *Lactobacillus, Bifidobacteria, Escherichia coli* (*E. coli*), *Enterococcus* and some yeasts (186).

Recently, supplementation with adequate probiotics has been shown to beneficially modify a number of major atherosclerosis-associated risk factors, such as hypercholesterolemia, dyslipidemia, hypertension, and chronic inflammation (187, 188). However, not all kinds of probiotics have been found to play protective roles in atherosclerosis. Chan et al. have demonstrated that supplementation with *VSL#3*, a consortium of eight lyophilized lactic acid bacterial strains, significantly reduced atherosclerosis lesion development of ApoE-/- mice induced by high-fat diet (189). Huang et al. found that the ApoE-/- mice that intervened with L.4356, belonging to *Lactobacillus* strains, presented a notable reduction in the atherosclerotic lesion area (190). One clinical trial showed that multispecies probiotics supplementation improved several parameters of endothelial dysfunction while Blattl et al. did not find some significant changes in 30 subjects with metabolic syndrome receiving *Lactobacillus casei* Shirota (191, 192). Several meta-analyses concluded that probiotics were associated with a significant reduction in TC and LDL-C, especially *Lactobacillus acidophilus* (193, 194). Although there is evidence that probiotics played some roles in host lipid profiling, the involved mechanisms are still not fully understood.

Besides, probiotics can improve the integrity of epithelial barriers and function of tight junctions which can effectively inhibit the harmful metabolite (TMAO, LPS, and so on) from entering into the circulatory system, proved to be able to prevent the progress of atherosclerosis (195). In the past, probiotics can make some beneficial effects on atherosclerotic risk factors, providing a promising therapy of atherosclerosis. However, little is understood about the mechanisms underlying the observed effects of probiotics on host health.

### Fecal Microbiota Transplantation

Recently, fecal microbiota transplantation (FMT) is popularized in the treatment of various systemic diseases related to dysbiosis of intestinal microorganisms (Figure 2). Up till now, in refractory and relapsed *Clostridium difficile* infection (CDI), FMT has already been shown to be effective and a primary therapy (196–198). In recent years, some scientists have found that FMT may become a potential approach for treating non-gastrointestinal diseases, such as treating CVDs, metabolic syndrome, diabetes, and so on. Being infused with intestinal microbes from lean donors, the obese patients can enhance insulin sensitivity known as the traditional risk factors for atherosclerosis (199, 200). In some mouse model experiments, FMT have already made some impressive benefits, such as the increasing production of SCFAs, extended lifespan, attenuated myocarditis, and so on (201, 202). However, some clinical trials have demonstrated that although FMT can effectively change the composition of gut microbiota, it did not show any practical influence such as TMAO production capacity, parameters related to vascular inflammation, and significant metabolic effects. Besides, the safety of FMT, a new therapeutical approach, is still unclear. First, it can disrupt the existing intestinal microorganisms, regardless of whether the bacteria are beneficial or damaging. Besides, FMT may transfer endotoxins or infectious agents into the circular system causing unnecessary complications (203, 204). Thus, more clinical trials and laboratory experiments are needed to study the efficiency of its application and its potential adverse events.

## Conclusion

In recent years, gut microbiota have been recognized as another organ of the human body. It not only affects the physiological processes of the host but also has been reported to be associated with several diseases such as cardiovascular diseases, endocrine disease, psychoses, and so on. In these diseases, CVDs were the most common underlying cause of death. In this review, we discussed whether the gut microbiota affects the stability of atherosclerotic plaque, whose rupture may cause a series of malignant events such as acute heart failure, myocardial infarction, and shock. Several studies have found that bacteria exist in atherosclerotic plaque, and there also exist some differences between the stable and unstable plaque. However, there is still no uniform conclusion on whether the bacteria will cause the plaque to more easily rupture or not. Besides, gut microbiota in the patient with atherosclerosis also show some changes compared with the control patient without atherosclerosis, and patients in diverse trials also present various alterations of the gut microbiota. Thus, it is still a long way for us to study the relationship between atherosclerosis and microbiota.

Except for the microbiota itself, the metabolites produced by gut microbiota also take various effects on the stability of plaque. As for TMAO, one of the metabolites, a great deal of studies detecting its influences on CVDs showed that it can provide more vulnerable characteristics to plaques. Besides, another metabolite, LPS, involved in infectious diseases has recently shown to have some effects on atherosclerosis based on in experimental and clinical trials by binding to various proteins, such as LBP, CD14, TLR-4, and MD-2. SCFAs, the main energetic resource of IECs, are able to maintain the gut barrier stable which can be the effective management to prevent the other harmful metabolites from entering into the circulation. SCFAs can prevent the development of atherosclerosis through traditional risk factors and VSMC proliferation.

Based on the characteristics of microbiota and its metabolites, there emerges a lot of new treatments to prevent plaque rupture and development of AS. Interference with lifestyle (healthy diet, supplying prebiotics, spending more time on exercise, some special drink or food), supplementation with probiotics, and FMT have recently been discussed for application in CVDs. However, due to the lack of long-term clinical trials, safety and effectiveness should still be explored.

## Author Contributions

XS conceived the topic and wrote the first draft. LL, ZS, GZ, LZ, CS, and ZW went through the manuscript, tables, and pictures. All authors revised and approved the final draft.

## Conflict of Interest

The authors declare that the research was conducted in the absence of any commercial or financial relationships that could be construed as a potential conflict of interest.

## Publisher's Note

All claims expressed in this article are solely those of the authors and do not necessarily represent those of their affiliated organizations, or those of the publisher, the editors and the reviewers. Any product that may be evaluated in this article, or claim that may be made by its manufacturer, is not guaranteed or endorsed by the publisher.
